# Electrical Resistivity Measurements of Surface-Coated Copper Foils

**DOI:** 10.3390/ma17122951

**Published:** 2024-06-17

**Authors:** Jiamiao Ni, Zhuoxin Yan, Yue Liu, Jian Wang

**Affiliations:** 1 State Key Lab of Metal Matrix Composites, School of Materials Science and Engineering, Shanghai Jiao Tong University, Shanghai 200240, China; nijiamiao@sjtu.edu.cn (J.N.); yan.77@sjtu.edu.cn (Z.Y.); 2 Department of Mechanical and Materials Engineering, University of Nebraska-Lincoln, Lincoln, NE 68588, USA

**Keywords:** surface-coated copper foils, four-probe method, electrical resistivity, surface damage

## Abstract

Due to the direct contact between the probe and sample, the contact of the four-probe method is important for the structural integrity of the sample and the accuracy of electrical resistivity measurements, especially for surface-coated metal foils with multilayered structures. Here, we analyzed the accuracy and stability of four-probe method probing on different sides of copper (Cu) foils covered with graphene (Gr). Theoretical simulations showed similar potential distributions on the probe tip when probing on the Cu and Gr sides. The resistivity of the Gr/Cu foil was 2.31 ± 0.02 μΩ·cm when measured by probing on the Cu side, and 2.30 ± 0.10 μΩ·cm when measured by probing on the Gr side. The major difference in the mean deviation is attributed to surface damage. In addition, the method of probing on the Cu side was sensitive to the resistivity changes of Gr induced by polymers with a dielectric constant range of 2~12, which is consistent with the calculations based on the random phase approximation theory. Our results demonstrated that the probing position on the metal side in the four-probe method can effectively protect the structural integrity of the functional surface-coated layer and maintain the high sensitivity of the measurement, providing guidance for the resistivity measurements of other similarly heterogeneous materials.

## 1. Introduction

The functional surface treatment of metal foils has been widely employed to improve the properties of metal foils, such as anti-oxidation [1,2], corrosion resistance [3,4], and electric resistivity [5,6]. However, their complex structures pose a significant challenge in measuring the electrical resistivity of these advanced conductive metal foils. Especially for metal foils with multilayered and easily damaged structures, the electrical resistivity measurement can easily introduce structural damage and large measurement errors during the test [7,8], which is not conducive to the associated studies. For example, the graphene/copper (Gr/Cu) foil has a typical layered structure, and the high-speed electron transmission layer of Gr could contribute to the ultra-high electrical conductivity of the Cu foil [9,10,11]. However, the probe tip may damage the layered structure of Gr/Cu foils because of the thin and heterogeneous Gr on the Cu foil. Correspondingly, the damage may greatly disturb the accuracy and reliability of the electrical resistivity measurement of Gr/Cu foils. It is therefore important to improve the reliability of resistivity measurements of such structural materials.

Generally, the methods for measuring the electrical resistivity can be classified into contact and non-contact measurements. Non-contact measurements are mainly based on the effect of conductor materials on electromagnetic fields, such as the eddy-current method [12,13] and high-frequency measurement [14]. However, due to the disturbance of the bottom and interface in the induced electric field in samples with ultra-low thicknesses and multilayered structures, the resistivity measured by these methods will have a large deviation [15,16]. As for other non-contact measurement methods like terahertz time-domain spectroscopy, electrical resistivity is measured based on the light excitation of the electronic response inside different energy bands and mainly focused on semiconductors [17,18,19,20,21]. The strong absorption of several metal materials in the spectrum, such as Cu, would greatly increase the difficulty of result analysis [22]. Contact measurement can be conducted using two-probe, three-probe, four-probe, and van der Pauw methods. Among these methods, the four-probe method is categorized as a universal method because of its high precision and simple process [23,24]. Here is the testing protocol: four probes (the outer two probes for the current source, and the inner probes for the voltage source), arranged horizontally at equal intervals, are used to measure the electrical resistivity of the sample. The large internal resistance of the voltmeter can effectively reduce the effect of contact resistance on the measured results. However, the probe tips in this contact measurement can easily damage the samples during measurement. Furthermore, due to the heterogeneous structure of surface-coated metal foils, the sensitivity and reliability of the measurement results may be influenced by different probe positions using the four-probe method. Therefore, the optimization of the contact in the four-probe method is essential to improve the accuracy of the results.

In this work, we compared the accuracy and sensitivity of four-probe method probing on different sides to measure the resistivity of surface-coated metal foils. We applied Gr/Cu foil as an example and probed on different sides of the foil. Combined with COMSOL simulation and measurement results, we found that probing on the Cu side can effectively avoid damage to Gr and maintain the stability of the measurement. And the sensitivity of probing on the Cu side was verified using Gr/Cu foils regulated by different coatings with dielectrics on Gr.

## 2. Materials and Methods

The Gr/Cu foil was fabricated using chemical vapor deposition (OTF-1200X-II-PE-RRW, Hefei Kejing Material Technology Co., Ltd., Hefei, China). The Cu foil was annealed at 1000 °C with 200 sccm H_2_ for 1 h to remove the oxidations on the Cu foil and improve the quality of Gr deposited on the Cu surface. After the annealing process, Gr was deposited on the Cu surface at 1000 °C with 6 sccm CH_4_ and 200 sccm H_2_ for 30 min. The pressure for annealing and growth was kept around 1 Torr. Then, the Gr/Cu foil was cooled down to room temperature in H_2_. As for the polymer-coated Gr/Cu foil, several kinds of polymers were dissolved in the solutions for 12 h at 70 °C: 5 wt% poly (vinylidene fluoride) (PVDF) in N, N-Dimethylformamide (DMF), 5 wt% polyvinyl alcohol (PVA) in deionized water, 5 wt% poly (methyl methacrylate) (PMMA) in DMF, and 5 wt% polystyrene (PS) in DMF. The polymer-dissolved solutions were coated on Gr/Cu foils respectively and then dried at 80 °C under Ar for 12 h.

The dielectric constants (κ) of these polymers were calculated by capacitance–frequency (*C-f*) curves and measured using the H2838 Series Precision LCR meter (Tonghui Electronics Co., Ltd., Changzhou, China). The top electrode of the measured structure is the Pt film deposited by magnetron sputtering, and the bottom electrode is the Gr/Cu foil. The value of κ can be calculated by $\kappa =Ct/A\varepsilon_{0}$, where *t* is the thickness of the coated polymer, *A* is the area of the top electrode, and $\varepsilon_{0}$ represents the dielectric constant of vacuum. The microscopic morphology of Gr was characterized using optical microscope (Axioscope 5, Zeiss, Oberkochen, Germany) and scanning electron microscope (SEM, RISE-MAGNA, TESCAN, Brno, Czech Republic). Raman spectroscopy (inVia Qontor, Renishaw, Gloucestershire, United Kingdom) was used to analyze the quality of Gr, and the laser wavelength was 532 nm. The four-probe measurement was conducted using a Cycle-Test high precision four-probe stage and Keithley 2460 instrument (Cleveland, OH, USA). The sample size was 1 cm × 1 cm. The current and potential fields inside the sample under four-probe measurements were simulated with COMSOL. The terminal potential of the current probe was set as 5 V.

## 3. Results and Discussion

**Figure 1:**
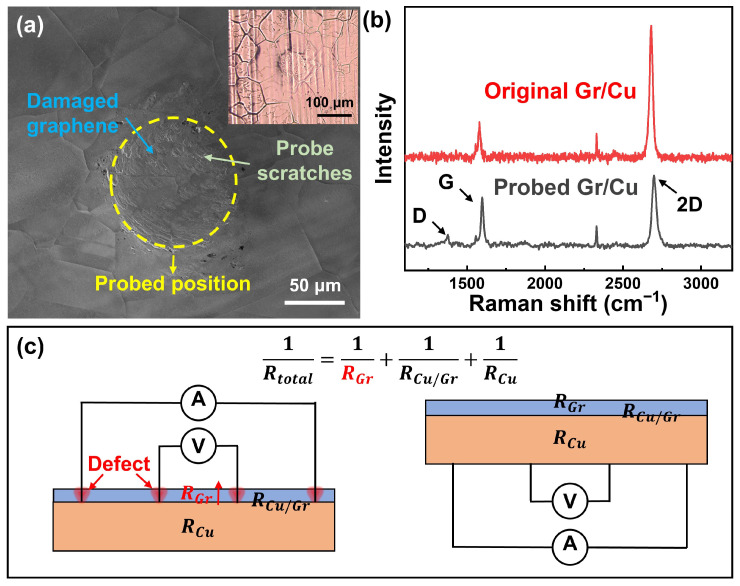
The effect of four-probe measurements on Gr/Cu foil. (**a**) The SEM image of the probed position on the Gr/Cu foil. The yellow circle indicates the probed position. The inset image shows the optical image of the probed position. (**b**) The Raman spectrum of the Gr/Cu foil before and after probing. The arrows mark the characteristic peaks of Gr (D peak, G peak, and 2D peak). (**c**) The schematics of the four-probe method with probing on the Gr side (**left**) and Cu side (**right**) in reality.

Firstly, the surface state of probing on the Gr side was performed to analyze the quality of surface-coated Gr in the Gr/Cu foil. As shown in Figure 1a, a visible pit and scratches appeared in the probed position on the Gr/Cu foil. The inhomogeneous contrast difference in the probed position indicates the separation and damage of the Gr, as shown in the SEM image of Figure 1a. Furthermore, the Raman spectrum of the probed Gr/Cu in Figure 1b also showed a defect peak (D peak, ~1350 cm^−1^) of Gr at the probed position, and the 2D peak (~2750 cm^−1^) was lower than that of the original Gr/Cu. This illustrates the destruction of quality and the layer structure of Gr in the probed region [25]. Corresponding to the layered structure of Gr/Cu foils, the electrical resistance of the Gr/Cu foil (*R_total_*) can be considered as the parallel resistance of the Gr resistance (*R_Gr_*), the contact resistance between Gr and Cu (*R_Gr_*_/*Cu*_), and the Cu resistance (*R_Cu_*). As shown in Figure 1c, the damages in Gr may contribute to the increase in *R_Gr_*. Consequently, the measured *R_total_* will deviate from the true value. In contrast, probing the Gr/Cu foil on the Cu side can effectively avoid detection on the Gr layer and maintain the intrinsic structure of Gr, as shown in the right schematic image of Figure 1c.

**Figure 2:**
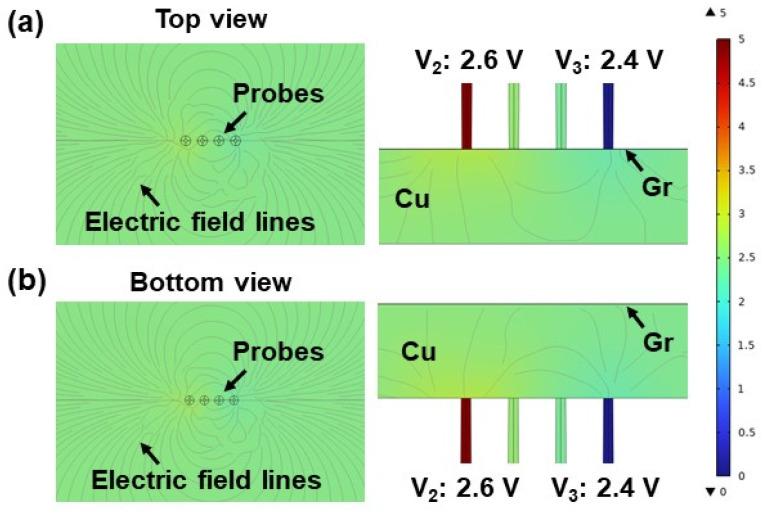
The electric fields of the different contact methods in COMSOL simulations. (**a**) The COMSOL simulation of the four-probe method with probing on the Gr side. The electric field lines in the right image show spherical symmetry in Gr/Cu. The potential difference *V_23_* is 0.2 V. (**b**) The COMSOL simulation of the four-probe method with probing on the Cu side. The electric field lines and *V*_23_ in this case are consistent with the results in (**a**).

To verify the influence of different detections on the measurement results, COMSOL simulations under four-probe methods with different contacts were performed to visualize the electric potential fields in Gr/Cu foils. According to the electric field lines in Figure 2 (the black lines inside the sample), the electric field lines generated by a point current source probe exhibit spherical symmetry in the Gr/Cu foil. The equipotential surface inside the sample is a hemisphere with radius *r*, demonstrated by the color distribution in Figure 2. Therefore, the electric field strength *E* and potential *V* were calculated as follows [26]:

(1) $$E=j\rho =\frac{I\rho }{2\pi r^{2}}$$

(2) $$V\left(r\right)=\int_{\infty }^{r}-Edr=-\frac{I\rho }{2\pi }\int_{\infty }^{r}\frac{dr}{r^{2}}=\frac{I\rho }{2\pi r}$$

where j is the density of the current. *R_total_* can be calculated according to the difference of the potential between probe 2 and 3:

(3) $$R_{total}=\frac{V_{2}-V_{3}}{I}=\frac{2\pi SV_{23}}{I}$$

where *S* is the distance of the probes. Considering that the size of the actual sample does not satisfy the semi-infinity condition [27], it is necessary to introduce the sample geometry coefficient *F* for correction based on Equation (3):

(4) $$R_{total}=\frac{2\pi SFV_{23}}{I}$$

According to the potential simulation results of the four-probe method with the different contacts, the potential difference *V*_23_ is 0.2 V for different methods. Therefore, the measured *R_total_* on the Cu side should be consistent with that measured on the Gr side, guaranteeing reliability from the point of view of the measurement principle.

**Figure 3:**
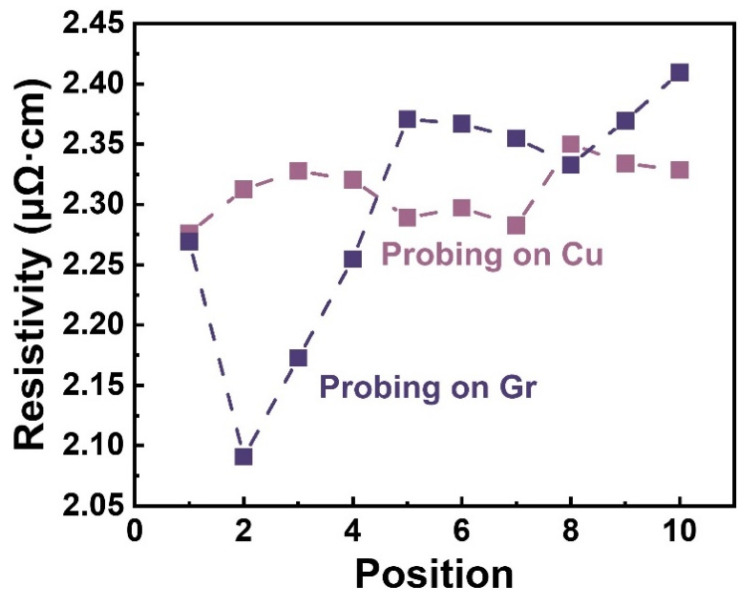
The measured resistivity of Gr/Cu foils with different contact methods. The result variance of probing on Cu is ~0.02 μΩ·cm, while the result variance of probing on Gr is ~0.10 μΩ·cm.

However, the damage in the Gr layer induced by the probes during the measurement may disturb the potential distribution and correspondingly affect the potential difference *V*_23_. Figure 3 compares the electrical resistivities measured on the Cu and Gr sides. Across 10 measurements at different positions, the resistivities of Gr/Cu foils measured by probing on the Cu side exhibited smaller deviations than those on the Gr side. The measured resistivities when probing on the Cu side had an average of 2.31 μΩ·cm with an error of ±0.02 μΩ·cm, while the results probing on the Gr side had an average of 2.30 μΩ·cm with a big error of ±0.10 μΩ·cm. These results suggest that the damage on Gr brings a larger fluctuation than the probe scratches on the Cu foil.

**Figure 4:**
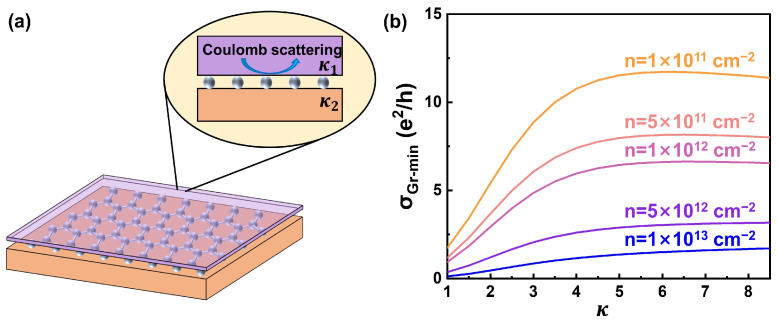
The effect of dielectrics on the electrical conductivity of Gr. (**a**) The schematic image of the dielectrics/Gr/Cu structure. The enlarged image demonstrates the Coulomb scattering from dielectrics to Gr. (**b**) The influence of the dielectric constant of the dielectrics on the $\sigma_{Gr-min}$ of Gr on the Cu foil, with different carrier density (n) of Gr.

Furthermore, we examined the effect of monolayer Gr on the electrical performance of the Cu foil by using the four-probe method with probing on the Cu side to confirm the sensitivity of this method. To avoid the interference of the inhomogeneity and structure-destroying caused by the substitutional doping of dopant atoms [28], we applied several coatings with different dielectric constants to regulate resistivity [29,30,31]. In general, the electron transport behaviors of intrinsic Gr are determined by the scattering of charged impurities from the substrates or other layers [29,32], as shown in Figure 4a. These charged impurities will contribute to the inhomogeneities of the carrier, resulting in the resistivity of Gr at the Dirac point. The minimum conductivity of Gr ($\sigma_{Gr-min}$) can provide feedback on the effect of the dielectrics on the electrical performance of Gr [32]. According to the self-consistent random phase approximation–Boltzmann formalism, $\sigma_{min-Gr}$ can be calculated by Equations (5) and (6):

(5) $$\sigma_{Gr-min}=\frac{{2e}^{2}n^{*}}{hn_{imp}G\left[2r_{s}\right]}$$

(6) $$\frac{G\left[x\right]}{x^{2}}=\frac{\pi }{4}+3x-\frac{3\pi x^{2}}{2}+\frac{x\left(3x^{2}-2\right)arccos\left[\frac{1}{x}\right]}{\sqrt{x^{2}-1}}\;$$

where *e* is the electronic charge, *h* is Planck’s constant, *n_imp_* is the concentration of charged impurities, and n* is the residual carrier concentration of Gr. The value of $n^{*}/n_{imp}$ can be obtained by Equations (7)–(9).

(7) $$\frac{n^{*}}{n_{imp}}=2r_{s}^{2}C_{0}^{RPA}\left(r_{s},\;a\right)$$

(8) $$r_{s}=\frac{2e^{2}}{\hbar \nu_{F}\left(\kappa_{1}+\kappa_{2}\right)}\;$$

(9) $$C_{0}^{RPA}\left(r_{s},\;a\right)=-\frac{1}{2r_{s}+1}-ln\left[\frac{2r_{s}}{2r_{s}+1}\right]-\frac{4ln\left(\bar{\gamma }a\right)}{{\left(2+\pi r_{s}\right)}^{2}}\;$$

where *r_s_* is the ratio between the coulomb potential energy and the kinetic energy in Gr, related to the dielectric constant of the substrate and surface coating dielectric of Gr ($\kappa_{1}$ and $\kappa_{2}$). A higher *r_s_* represents more Coulomb scattering from the substrate and dielectric to Gr. $\nu_{F}$ is the Fermi velocity, which is taken as 0.9 × 10^6^ m/s [33]. $C_{0}^{RPA}\left(r_{s},\;a\right)$ is the function of voltage fluctuation on *r_s_* and a, where $a=4k_{F}d$. d is the distance of charged impurities from the graphene layer and can be typically equal to 1 nm. $k_{F}=\sqrt{\pi n}$ is the Fermi wave vector. $\bar{\gamma }$ is Euler’s constant (~1.781). Therefore, the $\sigma_{Gr-min}$ of Gr on the Cu foil (κ=1) and other dielectrics with different κ can be calculated based on the carrier concentration (*n*).

As shown in Figure 4b, it can be calculated that $\sigma_{Gr-min}$ increases with higher κ and then gradually remains the same, similar to the experiment results [34,35]. The value of κ corresponding to the threshold region (~4) and the effect of κ on $\sigma_{Gr-min}$ are more obvious at the lower carrier concentration, corresponding to the higher quality of Gr. On the other hand, for Gr with a higher carrier concentration (*n* > 5 × 10^12^ cm^−2^), the effect of κ on $\sigma_{Gr-min}$ is relatively smaller, but the value of κ corresponding to the threshold is higher. For Gr with a low electron concentration, the variation in $\sigma_{Gr-min}$ with increasing dielectric constant is larger but there is a threshold. Therefore, the dielectric properties of the dielectric layer on Gr can effectively influence the electrical conductivity of Gr.

**Figure 5:**
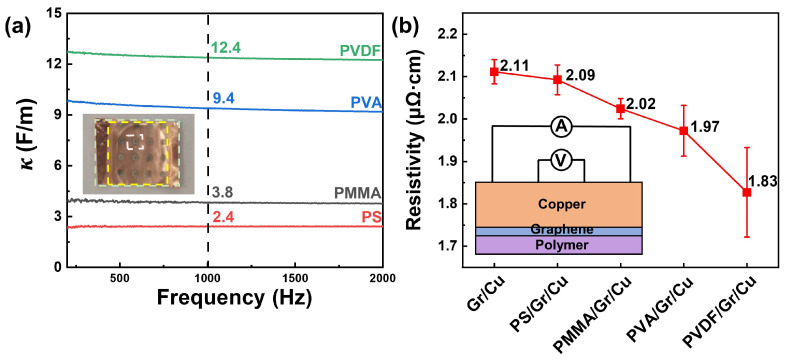
The results of different polymer/Gr/Cu foils measured using the four-probe method probing on the Cu side. (**a**) The dielectric constant of the different polymers measured by the capacitor structure. The inset image shows the measured structure: the top electrode is Pt, and the bottom electrode is the Cu foil. (**b**) The resistivity of different polymer/Gr/Cu foils measured using the four-probe method probing on the Cu side. The inset image shows the measured structure.

The resistivity of the Gr/Cu foil was then adjusted with polymers with different κ. The value of κ was measured with the capacitor structure, as shown in the inset image of Figure 5a. The dielectric constants of these polymers are as follows, in descending order: $\kappa_{PVDF}>\kappa_{PVA}>\kappa_{PMMA}>\kappa_{PS}$. Consequently, the resistivities of the polymer/Gr/Cu foils were measured using the four-probe method with probing on the Cu side, as illustrated in Figure 5b. The resistivities of these polymer/Gr/Cu foils are lower than that of the original Gr/Cu foil. The PS/Gr/Cu and PMMA/Gr/Cu foils that were modified by lower κ (2–4) polymers showed a significant and steady decrease in resistivity. As for the Gr/Cu foils modified with higher κ (>4) polymers, such as PVA/Gr/Cu and PVDF/Gr/Cu, the measured resistivities were further reduced due to the higher carrier concentration of vapor-deposited Gr on the Cu foil [36]. According to Newaz’s work [34], the polar properties induced by the high κ dielectrics will contribute additional scattering in Gr, which is different from the dielectric screening of the charged impurities and neglected by RPA. As a result, the non-uniformity of carriers in the PVA/Gr/Cu and PVDF/Gr/Cu is significant and contributes to a large error bar. The resistivities of Gr/Cu foils modified by different dielectric layers are consistent with the relevant theory, indicating the accuracy of the four-probe method when probing on the Cu side. In addition, the measurement fluctuations caused by the non-uniformity of the modified Gr/Cu foils also illustrated the high sensitivity of the measurements when probing on the Cu side. In addition to the electrical resistivity measurement of Gr/Cu foils, as for Gr composited with other metals, probing on the metal side which is not easily disturbed by the probes can also prevent damage to Gr and maintain high structural integrity and measurement accuracy. This strategy has great universality in the electrical resistivity measurement of materials with multilayered and easily damaged structures.

## 4. Conclusions

In summary, we used the four-probe method on different sides of a multilayered structure to reduce the disturbance of the probing damage on the electrical property. The resistivity of the Gr/Cu foil was measured using the four-probe method by probing on the two sides of the Gr/Cu foil. Due to the non-direct contact and less surface damage to Gr, the electrical resistivity measured by probing on the Cu side (2.31 ± 0.02 μΩ·cm) exhibited a smaller deviation than that measured by probing on the Gr side (2.30 ± 0.10 μΩ·cm). In addition, the method when probing on the Cu side was still sensitive to the resistivity changes of Gr induced by the polymers with a dielectric constant range of 2~12, which is consistent with the calculation based on the random phase approximation theory and the actual state of the sample. Our results demonstrated that probing on the metal side in the four-probe method can effectively protect the structural integrity of the functional surface-coated layer and maintain the high sensitivity of the measurement, providing guidance for resistivity measurements of other heterogeneous materials with a layer of fragile material. However, as for the heterogeneous structures with fragile materials in each layer, more effort needs to be focused on the optimization of the probe tips and the pressure during the test. This is the key factor to improving the accuracy of the contact measurement.

## Data Availability

The original contributions presented in the study are included in the article, further inquiries can be directed to the corresponding authors.
